# Evolution of fissures and pressure discharge of gas caused by mining of the upper protective layer of a coal seam

**DOI:** 10.1038/s41598-023-29020-1

**Published:** 2023-02-13

**Authors:** Honggao Xie, Xijian Li, Junjie Cai, Shengwei Wang, Cong Feng

**Affiliations:** 1 Mining College, Guizhou University, Guiyang, 550025 China; 2 School of Emergency Management and Safety Engineering, China University of Mining and Technology (Beijing), Beijing, 100083 China

**Keywords:** Energy science and technology, Engineering

## Abstract

The evolution of fissures and permeability associated with mining of the upper protective layer of the coal seam is crucial for pressure relief gas drainage of the underlying seam. To understand the influence of mining the upper protective layer on gas drainage within the underlying coal seam, this study utilized the M16 and M18 seams in the Qinglong Coal Mine in Guizhou. Theoretical analysis, discrete element numerical simulation, and field tests were used to characterize the evolution of fractures associated with mining of the upper protective layer and the effects of pressure relief gas drainage within the protected coal seam. The results show that mining-related stress changes controlled the development of fractures, altering the permeability values of coals. An analysis of the crack development in the coal mass caused by mining of the upper protective layer shows that during the initial stage of mining, the produced cracks exhibited a butterfly shape network. Yet, with further development of the mining, these cracks and the stress changes gradually produced an inverted butterfly shape network. According to simulations, the areas of maximum deformation via expansion in the protected coal seam were located near the open cut and the mining end line of the working face. The maximum deformation values were 29.06 and 26.68 mm, respectively, and the corresponding deformation rates were 9.37‰ and 8.61‰, which are greater than the required 3‰. The findings of this study provide a new reference for gas control in pressure relief coal seams under similar working conditions.

## Introduction

Coal is a major source of energy in China, > 85% of which is obtained from underground mines. Owing to its exploitation, approximately 33% of gas outbursts in the world occur in China^1–3^. Mining protective layer is divided into upper protective layer and lower protective layer. In order to eliminate the outburst danger of adjacent coal seams, the coal seams or rock strata mined first are called protective layers; the protective layer above the outburst dangerous coal seam is called the upper protective layer, and the lower protective layer is called the lower protective layer. Due to the mining action of protective layer mining and gas extraction at the same time, the outburst danger area of adjacent coal seam can be changed into no outburst danger area, which is called the protected layer. According to many scholars’ experience in gas control for many years, it is considered that protective layer mining technology is an effective technical means for gas control within the outburst coal seam^4–6^. The concentration of gas in the mine area gradually increases during underground mining, and the accumulation of this gas can eventually cause an outburst. Unlike in single coal seam mining, close mining of a coal seam associated with gas outburst is challenging. Owing to the complexity of the stress evolution in the unexcavated stage, accidents often occur during mining^7–9^. Therefore, to mitigate the accumulation of gas in the mine area and the occurrence of disasters underground, it is important to investigate gas drainage in the pressure-relieved coal seam that is associated with mining of the protective coal seam.

Generally, the foremost-mined coal seam is preferred that have no or less outburst hazard, and the adjacent rich-gas coal seams relieved the stress to increase permeability when mined the foremost-mined coal seam^10,11^. After coal mining, the stress of coal seam affected by mining changes, the coal body is destroyed. In this way, the coal seam fissures in the stress-relief area are enlarged, connecting the gas migration channel and increasing the permeability of the coal seam. The evolution of fissures and associated permeability changes caused by mining of the protective coal seams are vital for the control of gas outbursts during coal mining. To elucidate the mechanism of gas drainage in pressure-relieved coal seam caused by mining of the protective coal seam, experimental, theoretical, and numerical simulation studies have been conducted. Yuan and Xue^12^ used experiments involving the gas contents of coal seams to propose a method for the elimination of gas outbursts based on the mining extent of the protective coal seam. Li and Li performed experiments on interactions between the matrices of coals and cracks, and used the results to establish a coal permeability model. The elastic modulus associated with deformation caused by adsorption was also calculated because of the indirect effect of expansion from adsorption on permeability^13^. Further, Cheng et al. conducted an experimental study on the roof movement characteristics associated with stress-induced during close double-mining of a group of coals. The obvious discontinuity of cracks in the overlying strata was attributed to changes in the rock seam displacement fields that had resulted from mining disturbance^14^. In addition, the relationship between gas migration and coalbed methane enrichment was highlighted, and this was used to optimize the layout of holes for coalbed methane extraction, which improved extraction efficiency^15^. Gao et al. also indicated that fully developed fissures in coal associated with low stress from mining of the protective coal seam can enhance the permeability and stability of coal seams, and these improvements are beneficial for the extraction of gas, as well as for mining safety and efficiency^16^.

Regarding theoretical analyses, Yuan explored the extraction of gas via pressure-relief mining for varying coal (rock) layers and geological conditions, proposing an approach for the extraction of gas through pressure-relieved coal seam mining and gas. An innovative mine design for safe and efficient mining of low permeability and high gas-containing coal seams was also advanced^17^. Zhang et al.^18^ investigated the relationship between the migration of gas and its enrichment in fissures caused by mining, and proposed a zoning model of gas migration channels. Ni et al.^19^ establishment of mathematical model for crack length, construction of mathematical model for productivity using stage-fractured horizontal wells. Yang et al.^20^ used stress–damage–flow model to investigate the deformation and fracture characteristics of overburden strata, the evolution of gas permeability and gas flow in target coal seams. Zhou et al. then established a gas flow model under coal deformation that is associated with the Knudsen number (Kn)^21^.It was determined that, owing to changes in the apertures of cracks caused by the available stress and pore pressure, the transition among the Kn of methane and the gas flow pattern can also change. Wang et al. reviewed studies on protective layer mining technology and noted that rational planning based on existing conditions remains the principal approach for the prevention of gas outbursts during coal mining in China^22^.

In numerical simulations, Liu et al.^23^ indicated that the non-uniform distribution of the permeability of coal and gas pressure significantly influences the drainage of gas. Zhang et al.^24^ also used simulation to determine that for close coal seams containing high amounts of gas, mining of the protective layers can relieve pressure, thereby promoting the development of cracks that act as channels for gas migration. Chen et al.^25^ used simulation based on mining of a soft-rock protective coal seam to design a pressure-relieved coalbed gas drainage system featuring a pipe in the goaf as the core.

Therefore, existing studies have a significantly advanced understanding of mining of the protective coal seam and pressure-relieved gas drainage. Consequently, in the present study, gas pre-drainage effects associated with the drilling of holes in the M18 coal seam in the Qinglong Coal Mine in Guizhou Province were investigated. In view of the general effect of gas pre-drainage under the complicated coal seam gas occurrence conditions in Guizhou, it is difficult to reduce the coal seam gas content. In this paper, theoretical analysis, numerical simulation, and field tests were utilized to establish stress–fracture–seepage model. According to the coal seam stress change and crack evolution law caused by the mining of the upper protective layer, the gas migration law of the pressure-relieved coal seam was revealed, and then the gas drainage method of the pressure relief coal seam was optimized to achieve the purpose of reducing the coal seam gas content and reducing coal and gas outburst.

## Constitutive stress–fissure–seepage model for pressure relief of coal seams

**Figure 1:**
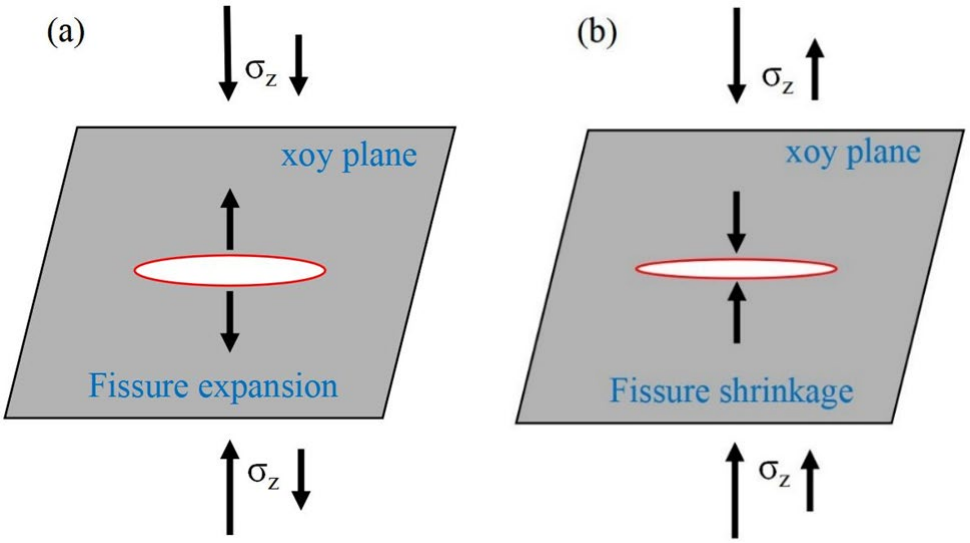
Schematic illustration of the relation between changes in a central horizontal fissure and stress in coals and rocks.

Typically, numerous primary fissures exist in protected coal and rock masses before mining. During mining, these fissures tend to contract or expand because of in situ stress changes. Figure 1a shows that if the normal stress (σ_z_) decreases, horizontal fissures may expand along the xoy plane, thus facilitating the horizontal migration of gas. Conversely, if the normal stress (σ_z_) increases, fissures in the xoy plane may shrink, thereby diminishing the horizontal gas flow as shown in Fig. 1b. The normal stress decisively impacts permeability changes in coals and rocks^26^.

According to Goodman and Bandis et al.^27–30^ the shrinkage or expansion of fissures in coals and rocks is significantly affected by stress, and the corresponding response determines permeability changes, which in turn also control the migration of gas associated with pressure relief. The relationship between the extent of the closure of a fissure and normal stress can be expressed as follows:

1 $$\sigma_{i} = \frac{{k_{i0} \delta }}{{1 - \left( {\delta /\delta_{m} } \right)}} = \frac{{k_{i0} \delta_{m} \delta }}{{\delta_{m} - \delta }}$$

where $\sigma_{i}$ is the normal stress (MPa), $k_{i0}$ is the initial stiffness of a fissure (N/m), $\delta$ is the extent of closure of the fissure (m), and $\delta_{m}$ is the maximum closure of the fissure (m).

Under normal stress, the extent of closure of a fissure can be expressed as follows:

2 $$\delta = \left( {\frac{{\sigma_{i} }}{{\sigma_{i} + k_{i0} \delta }}_{m} } \right)\delta_{m}$$

If b is defined as the fracture width (m), then *b* = *δ*_*m*_ − *δ*. According to Zhang et al. and Mathias et al.^31,32^ the permeability (*k*) associated with a fracture can be expressed as follows:

3 $$k{ = }\frac{{{\text{b}}^{{2}} }}{{{12}}}$$

Based on Eqs. (1, 2, 3) can be rewritten as follows:

4 $$k = \frac{{\delta_{m}^{2} }}{12}[1 - \frac{{\sigma_{i} }}{{\sigma_{i} + \sigma_{i0} }}]^{2}$$

where $\sigma_{i0} = k_{i0} \delta_{m}$ is the initial normal stress (MPa) and $\frac{{\delta_{m}^{2} }}{12}$ is a constant. The dimensionless permeability can be defined as $k_{f} = \frac{12}{{\delta_{m}^{2} }}k$, which can also be expressed as follows:

5 $$k_{f} = \left[ {1 - \frac{{\sigma_{i} }}{{\sigma_{i} + \sigma_{i0} }}} \right]^{2} = \left[ {\frac{1}{{\sigma_{i} /\sigma_{i0} + 1}}} \right]^{2}$$

**Figure 2:**
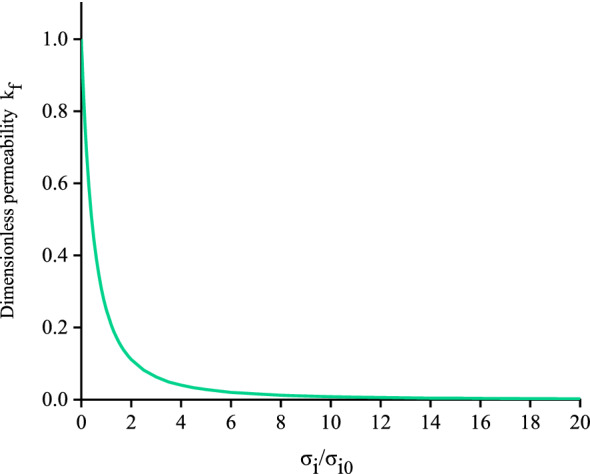
Plot showing changes in the dimensionless permeability as normal stress increases.

The relationship among the dimensionless permeability and normal stress of a coal seam based on Eq. (5) is shown in Fig. 2. As the normal stress increases, the dimensionless permeability decreases.

The normal stress on a coal seam is closely linked to its permeability, and the latter decreases as the former increases. Considering that a coal seam is exposed to stress in three dimensions, a three-dimensional (3D) constitutive stress–fissure–seepage pressure relief gas model (Eq. 6) can be established. According to the seepage model for a pressure-relieved coal seam, characteristics of the relieved gas drainage in the protected coal seam can be studied. These characteristics provide theoretical guidance for drainage of the pressure-relieved coalbed gas from the protected coal seam, as highlighted by the following expressions:

6 $$\left\{ {\begin{array}{*{20}l} {k_{f1} = \left[ {\frac{1}{{\sigma_{z} /\sigma_{z0} + 1}}} \right]^{2} } \hfill \\ {k_{f2} = \left[ {\frac{1}{{\sigma_{y} /\sigma_{y0} + 1}}} \right]^{2} } \hfill \\ {k_{f3} = \left[ {\frac{1}{{\sigma_{x} /\sigma_{x0} + 1}}} \right]^{2} } \hfill \\ \end{array} } \right.$$

where $k_{f1}$, $k_{f2}$, and $k_{f3}$ are dimensionless permeability notations along the xoy, xoz, and yoz planes, respectively, σ_x_, σ_y_, and σ_z_ are normal stresses along the x, y, and z directions (MPa), and σ_x0_, σ_y0_, and σ_z0_ represent initial normal stresses in the x, y, and z directions (MPa).

## Project example

### Project overview

**Figure 3:**
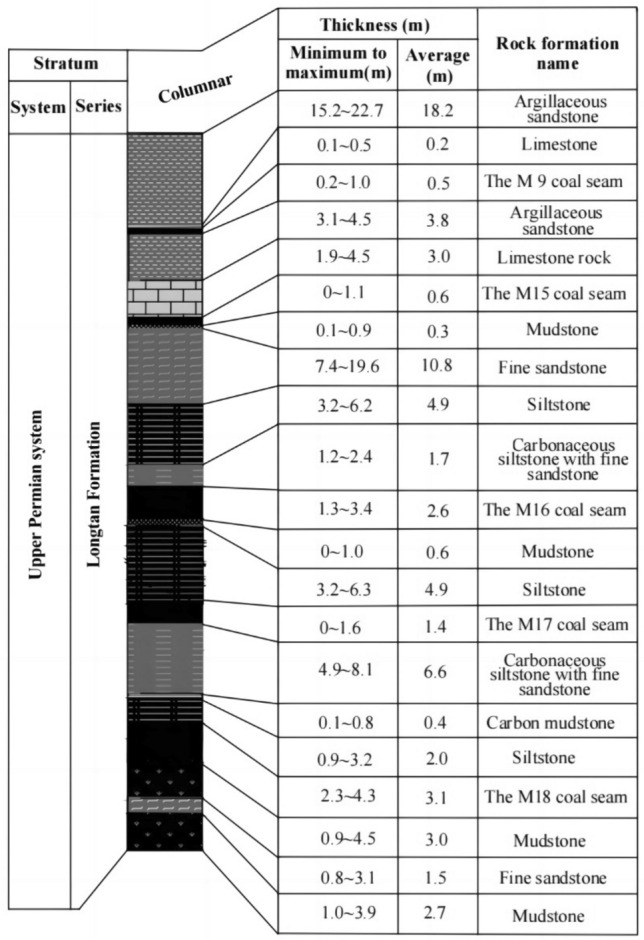
Geological section showing coal seams in the Qinglong mine district.

The Qinglong coal mine district, which is located in Guli in Qianxi County of Guizhou Province, China, has a geological structure of moderate complexity. Inclinations of strata in the mine district vary between 4° and 28° (commonly 9°–16°). Coal-bearing measures in the mine district comprise the middle and lower portions of the middle section of the Upper Permian Longtan Formation. The thickness of the formation ranges from 158.50 to 188.30 m, with the average value of 172.05 m. The coal-bearing layers vary in thickness from 15 to 26 m, and M16 and M18 are the principal minable seams, whereas M17 is locally minable coal. A vertical section showing the components of the Longtan Formation including coal seams is displayed in Fig. 3.

The buried depth of M16 coal seam in 21,605 working face is about 276–318 m. Dip angles of the coal seams vary between 9° and 16°.The working face strike length is 1550 m, the inclined length is 212 m, and the thickness ranges from 1.3 to 3.4 m, with an average mining height of 2.6 m. The M16 coal seam is estimated to contain between 12.61 and 18.28 m^3^ t^−1^ of gas, with an average of 15.62 m^3^ t^−1^, whereas the gas content of the M18 coal seam gas varies from 6.20 to 24.35 m^3^ t^−1^, with an average of 16.41 m^3^ t^−1^. The average permeability coefficient of the M18 coal seams is 7.1 m^2^ MPa^−2^ d^−1^, and both the M16 and M18 coal seams are characterized as soft, gas-rich, under high gas pressure, and exhibiting gas control challenges.

**Figure 4:**
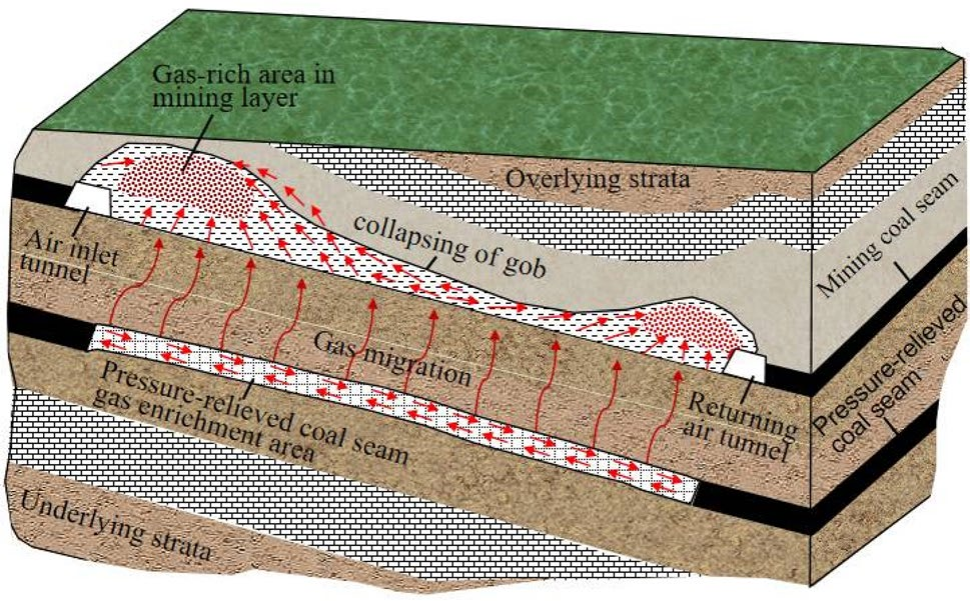
Conceptual illustration of the migration of gas caused by mining of the upper protective layer^33^.

The M18 coal seam gas was pre-drained via the 21,605 bottom drainage roadway, but the associated gas drained was insignificant. Therefore, mining of the upper protective layer was considered a means to solve the problem posed by its high gas content. Both the M16 and M18 coal seams exhibit potential for gas outbursts because the M16 coal seam is situated at an average height of 24.38 m above the roof of the M18 coal seam (12.25–40.50 m). Accordingly, considering that mining of the M18 coal will alter conditions in the M16 coal seam, and that the M17 coal seam is unstable and only partially recoverable, mining of the M16 coal seam (which is the upper protective layer of the M18 coal seam) is a reasonable approach. As shown in Fig. 4, after the coal seam is affected by mining pressure relief, the coal fissures expand, and the fissures are connected to form gas migration channels. The adsorbed gas in the coal seam desorbs into a free state, and then moves to the goaf to gather through these channels. At the same time, fissures will also be produced in the underlying coal seam to form gas migration channels, and the pressure relief gas will migrate or overflow to the mined-out area of the mining coal seam for enrichment.

## Analysis of gas pre-drainage in a cross-layer borehole

### Design of gas pre-extraction borehole

**Figure 5:**
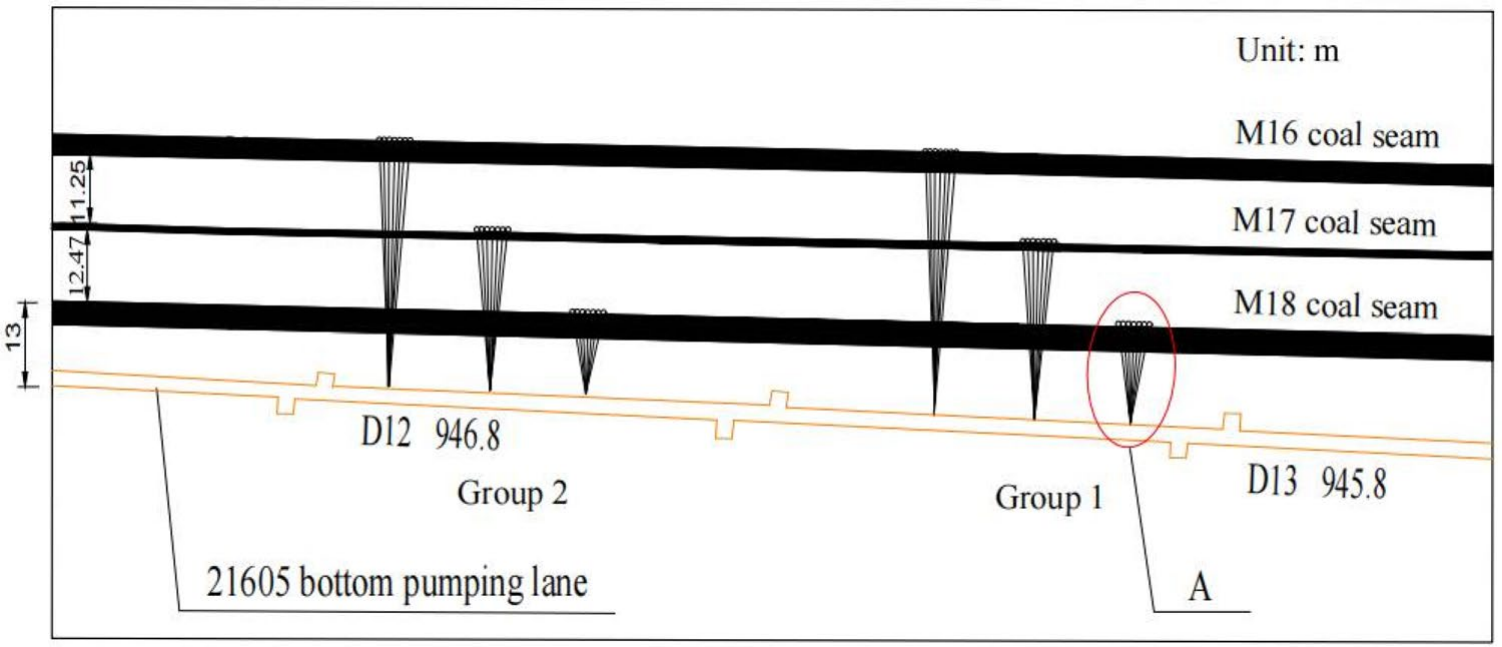
Diagram showing the layout of the 21,605 bottom pumping roadway channel.

**Figure 6:**
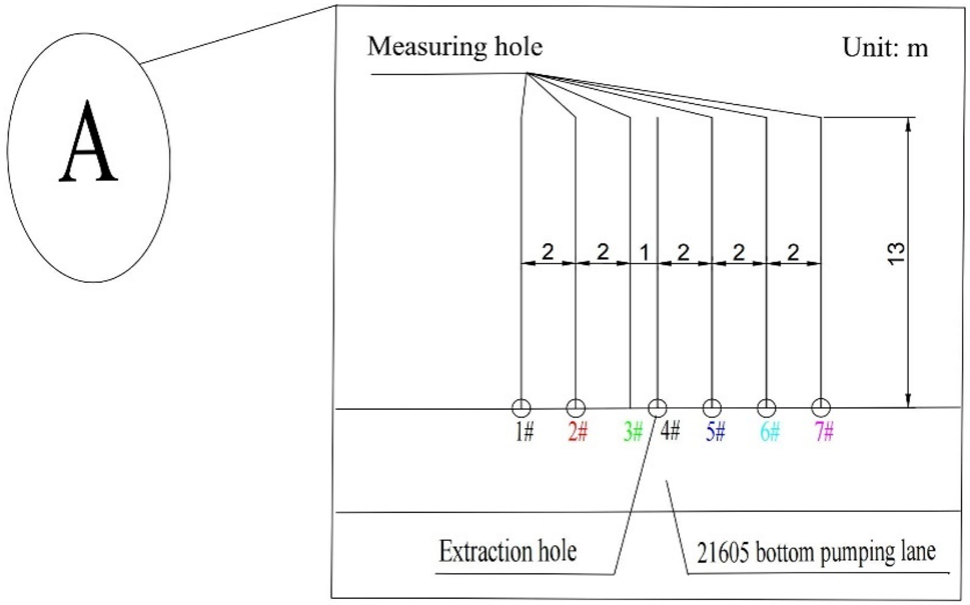
Design of the extraction and pressure measuring holes for the M18 coal seam.

Prior to mining that affected the M18 coal seam of the Qinglong Coal Mine, coal bed gas from this seam was pumped 20–210 m backward based on one control point (D13) in the 21,605 bottom pumping roadway via the Group 1 and 2 drill holes. As depicted in Fig. 5, the interval between the two sets of drill holes is 40 m. The “A”is M18 coal seam pressure measuring hole and drainage hole (see Fig. 6).

In Fig. 6, which shows that the 4# drill hole is for pumping, pressure relief holes were arranged at 1, 2, 3, 4, 5, and, 6 m from the extraction hole. The suction pressure was set to a value of 25 kPa, whereas the diameter of the borehole was 94 mm.

### Measurement and analysis of gas pressure

**Figure 7:**
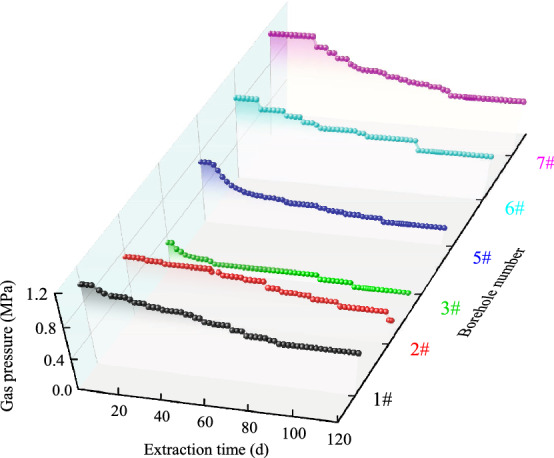
Plot showing the variation of gas pressure in cross-layer boreholes over time.

Gas was extracted for 120 d through the 4# drill hole, which is located 20 m from the control point (D13) of the 21,605 bottom pumping roadway. The variation trend of M18 coal seam gas pressure with the extraction time of the extraction process is shown in Fig. 7. The distances between drainage holes of pressure taps 1#, 6#, 7#, and 4# exceed 4 m. The pressure associated with gas in the three pressure taps obviously decreases before 80 d of drainage, and subsequently, pressure values stabilized to 0.55, 0.5, and 0.6 MPa for 1#, 6#, and 7#, respectively. Prior to 100 d of drainage, the pressure associated with gas at pressure tap 2# decreased gently, and subsequently, it settled at 0.3 MPa. Conversely, the pressure linked to gas at 5# dropped sharply before 20 d of gas extraction, and then stabilized at 0.21 MPa. Relatedly, the pressure associated with gas decreased from 0.31 to 0.05 MPa within 120 d of drainage at pressure tap 3#. According to the *Detailed Rules for Prevention and Control of Coal and Gas Outburst*^34^ the distance of a pressure measuring hole to an extraction hole when the pressure linked to gas decreases to < 0.74 MPa is the effective extraction radius. Figure 7 shows that gas pre-drainage near the drainage hole is significant within the effective drainage radius, whereas the impact diminishes when the drainage radius exceeds 4 m.

### Determination and analysis of the residual gas content

Original gas contents in six measuring holes in the M18 coal seam were measured via coring. After 120 d of extraction, the residual gas content was measured according to original drilling parameters at 0.5 m (numbered 1#, 2#, 3#, 5#, 6#, and 7# in turn), and the results are presented in Table 1. According to the *Detailed Rules for Prevention and Control of Coal and Gas Outburst*^34^, if the gas pressure is < 0.74 MPa, the gas extracted will be < 8 m^3^ t^−1^ (the gas content in the structural zone will be < 6 m^3^ t^−1^), thus to prevent a gas outburst, the gas extraction rate must exceed 30%. Data in Fig. 7 and Table 1 show that residual gas content in the 2#, #3#, and 5# coal samples was < 8 m^3^ t^−1^, and their gas pre-extraction rates exceeded 30%. This indicates that if the extraction radius is < 4 m, gas pre-extraction will be suitable for the prevention of an outburst. Conversely, the residual gas content of the 1#, 6#, and 7# coal samples was > 8 m^3^ t^−1^, and the corresponding gas pre-extraction rates were < 30%, and thus, gas extraction would not prevent a gas outburst.

**Table 1:** Summary of sample locations and the associated original and residual gas contents.

Number	Sample location in relation to distance from control point D13 of the 2605 bottom pumping roadway (m)	Azimuth (°)	inclination angle (°)	Original gas content (m^3^ t^−1^)	Residual gas content (m^3^ t^−1^)	Extraction rate (%)
1	15	90	90	9.8214	9.6542	1.70
2	17	90	90	11.3526	6.5802	42.04
3	19	90	90	10.8203	5.0963	52.90
5	22	90	90	12.0711	6.4478	46.58
6	24	90	90	10.0256	9.5258	4.99
7	26	90	90	12.2812	8.9304	27.28

The original and residual gas contents both involve non-desorption gas.

Therefore, according to analysis of the gas pre-drainage data for the M18 coal seam, if the drainage radius is < 4 m, the gas pressure and content in the area significantly decreases, and the effect of the process is good. Conversely, if the drainage radius is > 4 m, the gas pressure and content of intra-regional decrease slightly, and the gas pre-drainage effect is moderate. Considering the combination of high original gas content, complicated geological conditions of coal seam, and insignificant gas pre-drainage effect associated with the M18 coal seam, mining of the protective coal seam can be used, and the gas drainage drilling hole can be designed to control gas drainage from the M18 coal seam, so as to realize safe and efficient mining of coal and gas drainage of the pressure-relieved coal seam.

## Analysis of fissure evolution and pressure relief gas extraction

### Upper protective layer mining discrete element model

**Figure 8:**
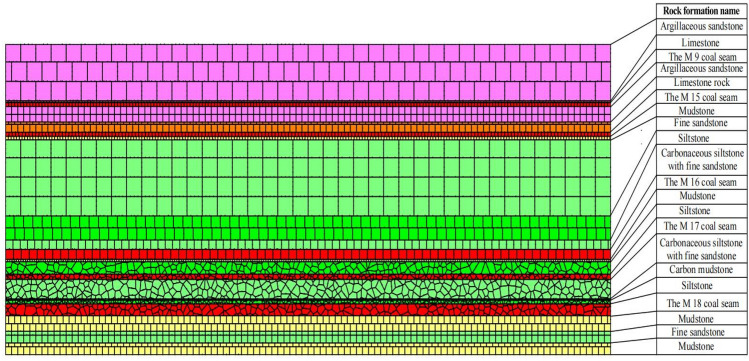
Schematic representation of the numerical model associated with mining of the upper protective layer.

Based on geological conditions at the 21,605 working face in the Qinglong Coal Mine, a UDEC model was established (Fig. 8). The model simulated the M16 coal seam, with the length, height, and mining height of 200, 80, and 2.6 m, respectively. Joints were grouped according to the lithology, and then the boundaries of the model were constrained and fixed. Concurrently, to minimize the effects of the left and right boundaries, 20 m coal pillars were retained on both sides. The mining length between the pillars was 160 m, and mining proceeded by covering successive 20 m intervals, whereas a load pressure of 7.5 MPa was used to simulate the weight of the overlying rock mass. The rock mass was considered to have elastic–plastic behavior and obey the Mohr–Coulomb model, and thus, the joint surface contact-Coulomb slip criterion was adopted^35^. Data for the physical and mechanical properties of the various rock types are presented in Table 2.

**Table 2:** Physical and mechanical property data of strata in the mine district.

Rock formation name	Density (kg m^−3^)	Modulus of elasticity (GPa)	Tensile strength (MPa)	Internal cohesion (MPa)	Angle of internal friction (°)	Poisson’s ratio
Siltstone	2700	19.5	1.8	2	30	0.15
Mudstone	2690	15	1.7	3.0	30	0.28
Carbon mudstone	2810	10	1.5	2.5	30	0.29
Coal	1400	19.5	0.5	1	22	0.15
Fine sandstone	2660	19.5	1.8	2.5	30	0.15
Limestone rock	2750	32	3	6	38	0.21
Muddy sandstone	2690	15	1.7	3.0	30	0.28
Limestone	2670	32	3	2.5	33	0.21

Data in the table were obtained from records associated with deep mines and the literature^36^.

### Evolution of fissures in the upper protective layer

**Figure 9:**
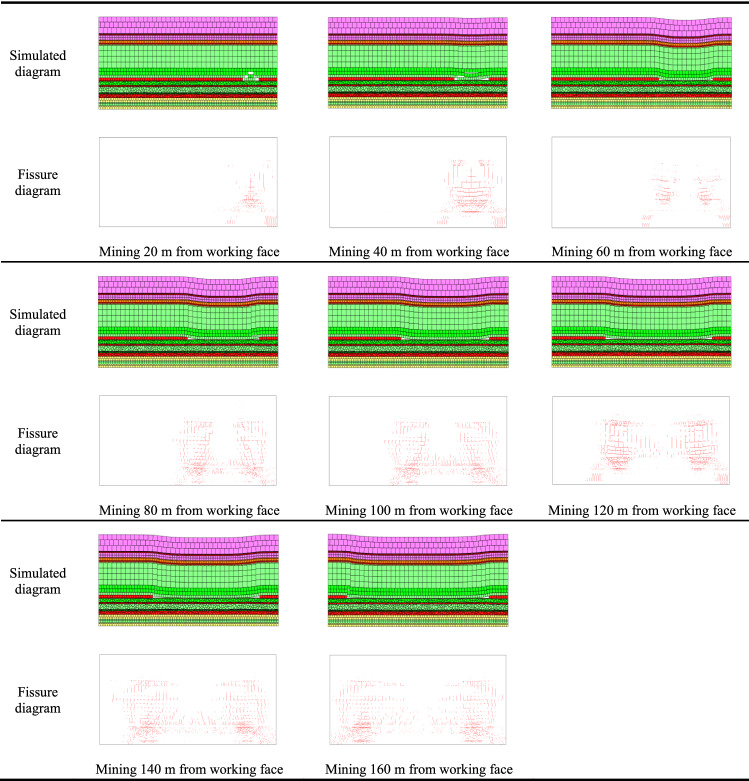
Evolution patterns of fissures in the protective layer based on mining at different distances from the initial working face.

The 21,605 working face was simulated by mining 20 m intervals. Outputs of the simulation and evolution of fractures in the vertical areas for the overlying and underlying rocks based on distances of 20, 40, 60, 80, 100, 120, 140, and 160 m from the original working face are shown in Fig. 9.

The coal matrix permeability of coal seams is low, and thus the development and distribution of cleat and fracture systems play a major role in CBM production^37^.The rock roof can be caved, fractured and deformed in the upward direction during underlying coal mining^38^.As shown in Fig. 9, mining 20 m from the initial working face produced cracks in the overlying and underlying strata of the M16 coal seam. As the underlying strata swelled and expanded, cracks in the vertical area of rocks evolved into a thin butterfly shape. At mining distances of 40–60 m from the initial working face, the main stratum (basic roof) bent and new fissures increased, and as the floor of the M18 coal seam expanded further, joints and fissures also widened. However, as the working face advanced, because of the contact between caving strata in the goaf and the underlying swelling coal seam, bulging of the seam floor was limited. Figure 9 shows that because of compaction induced by the collapsed rock stratum, the middle portion was characterized by a high fracture density, and pressure relief was obvious at both ends of the goaf. In the vertical zone of the stratum, the butterfly-shaped fracture network gradually spread from the middle portion to the sides. As the working face advanced to 60–80 m, because the peak stress in the surrounding rock was lower than that in the initial stage of mining, bulging of the floor was obvious. Consequently, the butterfly-shaped fracture area spread, and many cracks that were produced extended deeper. Relatedly, if the working face advanced to 80–120 m, the roof of the mined-out area collapsed, whereas strata in the middle were compacted. The expansion of the underlying strata was restricted, and cracks in the middle of the mined-out area mostly shrank and closed, and the development of new cracks was slow. These fractures gradually produced an inverted butterfly shape, which indicates that the direction of stress at some locations in the underlying rock changed. This is similar to what Wang et al.^39^ found the intermediate principal stress plays a key role in the process of wing crack growth. If mining was advanced to 120–140 m from the initial working face, the caving roof strata in the goaf became largely compacted, and cracks in the center of the floor strata shrank and closed. However, cracks continuously developed at both ends, and these were also characterized by an inverted butterfly shape. Regarding mining at 140–160 m from the initial working face, the development of cracks in the underlying strata seemed stable and was characterized by the gradual compaction of coal and rocks because of the load, thereby producing compaction-related cracks. Cracks at this point were mainly near the open cut and end of the mining line of the working face.

### Vertical expansion and deformation patterns of the protected layer

**Figure 10:**
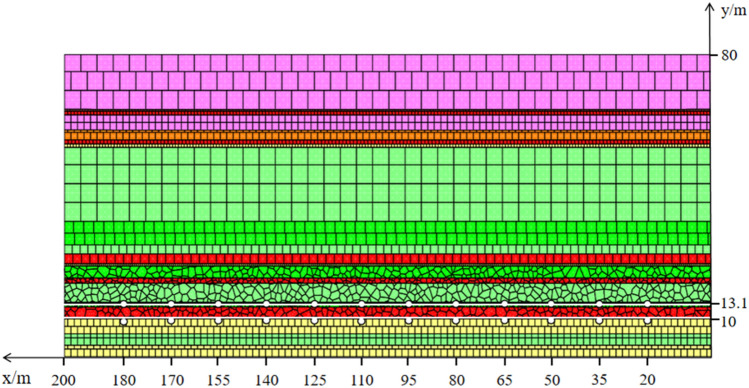
Layout of stress lines and measuring points for the M18 coal seam.

**Figure 11:**
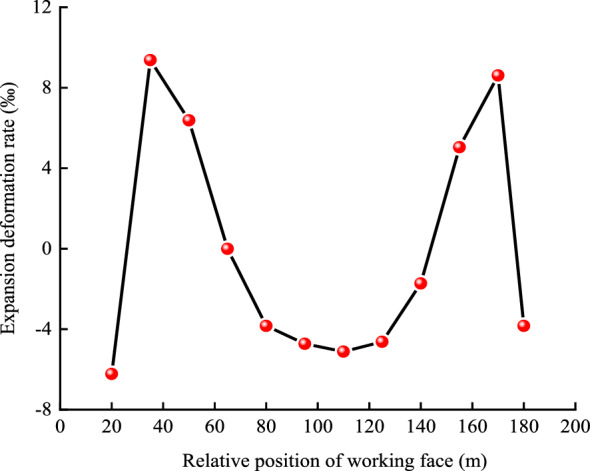
Plot showing the relationship between the deformation rate of the protected layer and the relative position of the strike of the working face.

To further understand the relationship between changes in the thickness of the protected layer and the mining distance from the initial working face, two survey lines were created on the roof and the floor of the protected layer. Each stress survey line began at 20 m from the right boundary and the point interval was 15 m, with the last point interval being 10 m. There were 12 points in total (Fig. 10). Changes in the thickness of the protected layer during the mining of up to 160 m of the protective layer are displayed in Fig. 11. The value for the first measurement site highlights compression of the M18 coal seam, but as mining proceeded, the coal seam gradually swelled. It reached a maximum deformation via expansion of 29.06 mm when mining was 35 m from the initial working face, and the associated deformation rate was 9.37‰. The thickness change values displayed in the middle section of the profile reveal that during mining between 65 AND 140 m from the initial working face, the M18 coal seam was compressed. The maximum compression was 16.30 mm, corresponding to a deformation rate of 6.23‰. At mining distances from 150 to 170 m, the coal seam resumed expansion, and the maximum expansion of 26.68 mm that was attained represents a deformation rate of 8.61‰. These results satisfy the requirement that the deformation rate of the protected layer should exceed 3‰ as stated in the *Detailed Rules for Prevention and Control of Coal and Gas Outburst*^34^*.* These results indicate that mining of the upper protective layer should adequately relieve gas pressure and enhance the permeability of the protected layer.

### Analysis of stress change in the protected layer

**Figure 12:**
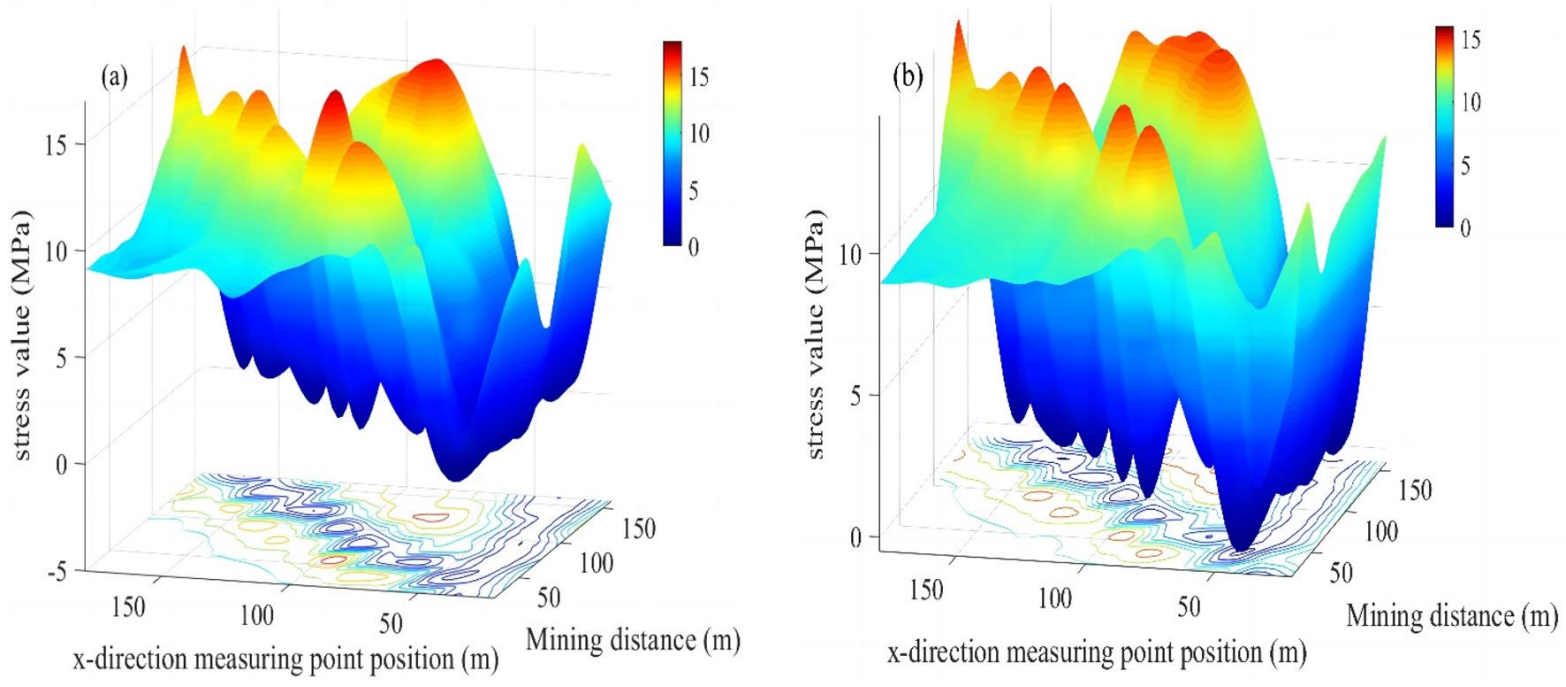
Models showing stress changes as a function of relative mining distance of the working face for (**a**) the roof and (**b**) the floor of the M18 coal seam.

The stress changes in the M18 coal seam caused by the mining of 20–160 m of the M16 coal seam, as measured using survey lines on the roof and floor, are presented in Tables 3 and 4, respectively. The Surfc function in Matlab was utilized to plot relationships between stresses in the roof and floor versus the mining distance (Fig. 12). These plots highlight low stress areas and relationships between the maximum normal stress and the advance of the working face.

**Table 3:**
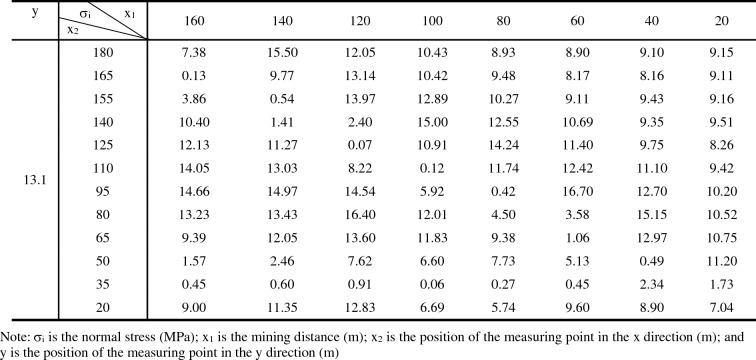
Data for stress changes in the roof of the M18 Coal seam during mining of the M16 coal seam.

σ_i_ is the normal stress (MPa); x_1_ is the mining distance (m); x_2_ is the position of the measuring point in the x direction (m); and y is the position of the measuring point in the y direction (m).

**Table 4:**
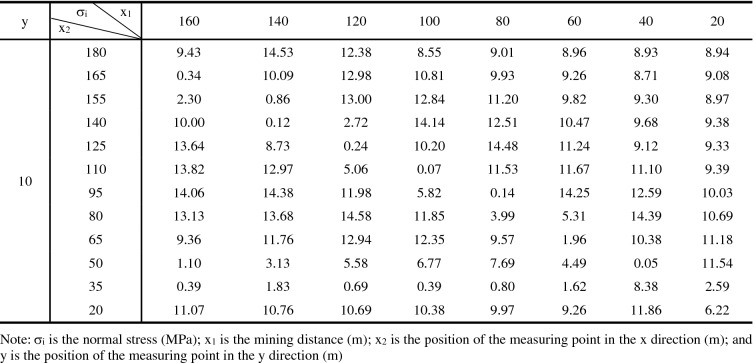
Data of stress changes in the floor of the M18 coal seam during mining of the M16 coal seam.

σ_i_ is the normal stress (MPa); x_1_ is the mining distance (m); x_2_ is the position of the measuring point in the x direction (m); and y is the position of the measuring point in the y direction (m).

Figure 12 demonstrates that if the relative working face is ≤ 20 m, the stress will be comparable to that before mining commenced. However, as the mining distance increases, a stress concentration area will emerge. Pressure in the coal seam after the relief was obviously lower than the initial stresses at measurement points on the goaf, and these areas are denoted as the high-strength pressure relief infiltration areas. Figure 12 also reveals that as mining advances, the stress will increase at measurement points in the goaf. The increased stress values are attributed to the collapse and recompression of strata in the goaf, and these are termed recompression areas.

**Figure 13:**
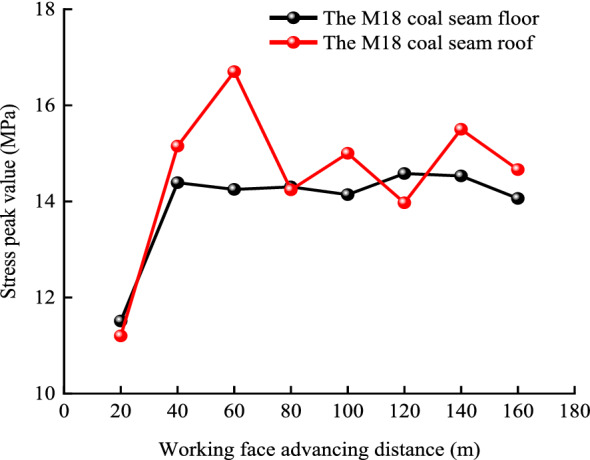
Plot showing stress changes along survey lines along the floor and roof of the M18 coal seam.

The relationship between the maximum normal stress peak value and the advancing distance of the working face is shown in Fig. 13. Figures 12 and 13 show that as the relative distance of the working face changed from 20 to 40 m, the peak stress in the roof and floor of the M18 coal seam increased. These results indicate that the main load layer is the roof strata in the goaf, and thus, a stress concentration phenomenon emerged in this area. However, as the stress in the area corresponding to mining distances of 21–39 m (termed the high-strength pressure relief permeability area) was lower than the initial stress, the expansion of fractures in that area enhanced the permeability of the associated strata. As the working face advanced from 40 to 80 m, the peak stress in the roof of the M18 coal seam reached its maximum at approximately 60 m. Relatedly, as the relative working face advanced to 80–100 m, the peak stress in the roof of the M18 coal seam decreased rapidly, highlighting stress variations, whereas the peak stress in the floor of the seam was stable. At this point, due to the collapse of the rock formation in the goaf, the coal formation was recompressed, and stresses in the roof and floor of the coal seam increased slightly above those measured at mining distances of 20 m because of recompression (this area is termed the recompression area). The coal seam at this point was exposed to loading, unloading, and reloading, and the rock mass was altered because of stresses that are higher than the initial stress, and thus, some cracks were closed. As the working face reached 160 m, stresses in the roof and floor near the corresponding measurement points at 180 m decreased, and permeability values in these areas increased obviously. The range of stress fluctuations in the roof and floor of the M18 coal seam were narrow, and thus, overall, stresses in these areas could be characterized as stable. The relative stability of stress in the coal seam is owing to the redistribution of stress associated with the large-scale caving of strata in the goaf. The pressure relief effects on the coal seam in the area near the open-cut and the end of the mining line of the working face were obvious.

### Drill holes design for pressure relief gas drainage of the protected layer

**Figure 14:**
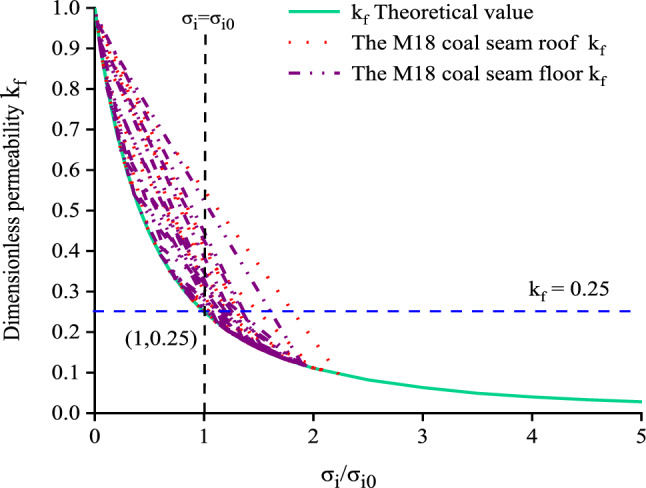
Variation of permeability as a function normal stress in the roof and floor of the M18 coal seam.

For the M18 coal seam roof and floor, k_f_ was calculated by Eq. (6) according to the measured stress of the measuring points on the two measuring lines of the M18 coal seam roof and floor when the working face was mined to 160 m, with data that are presented in Tables 3 and 4. As shown in Fig. 14, the permeability in the roof and floor of the M18 coal seam roof and floor line changed with the stress on the side line, and the permeability decreased with the increase of stress. Here, in the numerical simulation, the load exerted by the overlying strata is 7.5 MPa as the original stress.

When σ_i_/σ_i0_ = 1(the stress is equal to the original stress), dimensionless permeability kf = 0.25, when the forward stress σi decreases, the permeability increases, and dimensionless permeability kf > 0.25. However, in the case that the dimensionless permeability of some of the figures is greater than 0.25, this is because the stress in the recompacted area first decreases, then increases, and finally tends to equilibrium, and the cracks gradually shrink after propagation. At this time, the permeability will be slightly greater than the initial state, but less than the permeability in the hyperpermeability and permeability area of pressure relief. It can be judged that under the influence of coal seam mining and pressure relief, the coal seam permeability will increase, which is conducive to the diffusion of gas.

**Figure 15:**
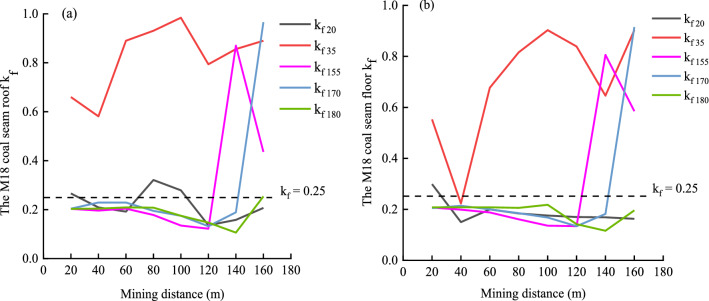
Plots showing relationships between permeability and the distance of mining from the working face for (**a**) the roof and (**b**) floor of the M18 coal seam.

Changes in permeability of the coal seam during mining were determined based on data for the roof and floor from Tables 3 and 4, as well as Eq. (6). As shown in Fig. 15a, after mining for 160 m, the permeability of M18 coal seam roof strata is the highest at 35 m (k_f35_) in the direction of mining face, and kf is much higher than 0.25. From Figs. 9 and 12, it can be found that the stress of this measuring point is small, and it is located in the densely mined fracture area. While the permeability of k_f155_ and k_f170_ near the goaf before the rock caving is higher when the mining face is advancing, and the permeability of k_f35_, k_f155_ and k_f170_ near the stop mining line is relatively higher. The reason is that after the protective layer is mined to the stop mining line, there are high-strength pressure relief seepage areas near the open cut and near the stop mining line (the range is about 15–20 m) because of the stress reduction. The permeability of other measuring points is mostly higher in goaf area. It can be seen from Fig. 15b that the change trend of coal seam floor permeability is basically similar to that of roof permeability. In addition, near the open cut, depressurization caused expansion of the coal seam, and the creation and opening of joints increased both the porosity and permeability. Consequently, abundant desorption and diffusion of the gas occurred, and the associated decline in pore pressure further enhanced the permeability.

**Figure 16:**
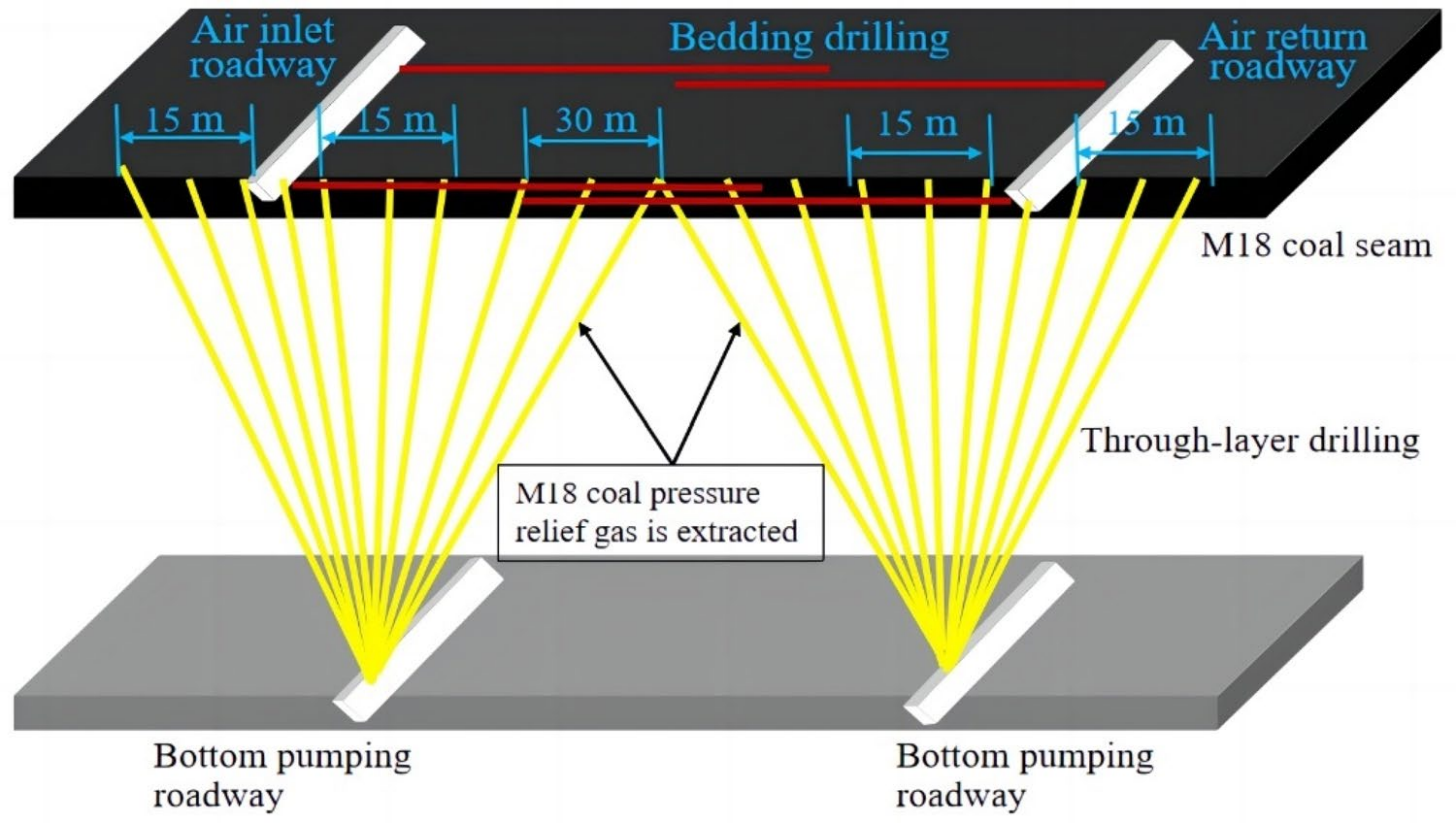
Diagram illustrating the design involving a pressure relief gas borehole in the protected layer.

Mining of the protective layer was concurrent with the extraction of gas from the protected layer through pressure relief, and thus, this minimized the risk of a gas outburst. In the mining of the highly-gassy coal seams, strengthening gas drainage in the goaf and fissure zone can improve the efficiency of coal seams and reduce carbon emissions^40^. According to the actual situation in the mine, pressure relief holes have been drilled vertically from the bottom pumping roadway to extract gas from the M18 coal seam, and the layout of these holes are depicted in Fig. 16. Two groups comprising ten holes were designed for drilling from the 21,605 bottom pumping roadway. Among these holes, seven were drilled at the ends of the mine which are characterized by high fracture densities. These holes were spaced at 15 m, whereas three holes were situated in the center of the coal seam, and these were spaced 30 m. The design involved a negative pressure of 25 kPa, a drill hole diameter of 94 mm, and a hole spacing of 15–30 m.

According to an onsite investigation, the permeability coefficient of the M18 coal seam increased from 7.1 to 101.2 m^2^ MPa^−2^ d^−1^, which represents an increase by a factor of 14.25. Owing to the pressure relief drilling, the gas extraction rate from the M18 coal seam in the protected area reached 72.41%. In fact, the gas content of the coal seam decreased from 9.6542 to 6.98 m^3^ t^−1^ and the gas pressure dropped from 1.6 to 0.47 MPa. According to the *Detailed Rules for Prevention and Control of Coal and Gas Outburst*^34^, mining of the M16 coal seam (protective layer) combined with the pressure relief gas extraction technology effectively reduced the risk of gas outbursts in the M18 coal seam. Therefore, safe and efficient mining conditions were created for the main coal seam M18.

### Ethics approval

All authors declare that all contents of this study are original.

## Conclusions

1. Prior to mining of the protective layer, primary cracks are usually present in coal and rock masses. Owing to mining, these strata are affected by changes in stress. These changes cause expansion and closure of cracks, which increase or decrease the permeability values of coal and rock masses. Considering that coal seams and rocks are under stress in three dimensions, the present study established a constitutive stress–fracture–seepage pressure relief gas model for a coal seam.
2. Analyze the development of cracks caused by mining of the upper protective layer in the underlying coal seam. In the vertical plastic zone of rocks, fractures that developed first exhibited a butterfly shape, but as mining advanced, stress variations caused cracks displaying an inverted butterfly shape. Owing to the mining of the protective layer, the in situ stress in the protected coal seam decreased, and this pressure relief caused expansion. The expansion created and widened the joints and fissures, increasing both the porosity and permeability. These changes were accompanied by the desorption and diffusion of abundant adsorbed gas. The pressure in pores decreased further, thereby increasing the permeability.
3. The maximum area of deformation via expansion of the protected coal seam was located near the open cut and the end of the working face (i.e., both ends of the mined-out area). The maximum deformation values were 29.06 and 26.68 mm, respectively, with corresponding deformation rates of 9.37‰ and 8.61‰. These deformation rates significantly exceed the recommended value of 3‰ for the prevention of gas outbursts, and these higher rates can be attributed to the mining of the protective layer.
4. These findings enabled optimization of the layout of holes for the pressure relief gas drainage. According to field investigation results, the permeability coefficient of the protected M18 coal seam increased from 7.1 to 101.2 m^2^ MPa^−2^ d^−1^, an increase factor of 14.25. The gas extraction rate from the M18 coal seam via the borehole pressure relief attained 72.41%. Both the gas content and the associated pressure decreased, thereby minimizing the chance of a gas outburst. The findings of the present study are useful as a reference for the control of gas through pressure relief in coal seams that are characterized by similar conditions.

This study is based on the principle that the gas pre-drainage effect in high-gas mines is not significant; our approach considered protective layer mining and optimization of the drilling position of the pressure-relief coal seam for extracting gas in order to achieve the safe mining of coal and gas. In this research, only the stress–fracture–permeability model of the coal seam under the influence of two-dimensional in-plane mining was studied. In future research, the stress changes in 3D space of the coal seam under the influence of mining will be investigated, and the factors affecting gas migration (e.g., gas pressure) will be taken into account in the model, which will be applied to the surface mining of coalbed methane with the aim improving the surface mining efficiency of coalbed methane.

## Data Availability

The data used to support the findings of this research are included within the paper.
